# Endothelial Cells Filopodia in the Anastomosis of Central Nervous System Capillaries

**DOI:** 10.3389/fnana.2019.00049

**Published:** 2019-06-03

**Authors:** Miguel Marín-Padilla, Louisa Howard

**Affiliations:** Department of Pathology, The Geisel School of Medicine at Dartmouth, Hanover, NH, United States

**Keywords:** capillary anastomosis, CNS capillaries, endothelial cell filopodia, filopodia conglomerates, new post-anastomotic CNS capillaries

## Abstract

In this article we explore filopodia of endothelial cells (ECs) in the developing central nervous system (CNS) using the Golgi method and transmission electron microscopy. Filopodia of ECs play a crucial role in the anastomosis of growing capillaries of the CNS. The leading ECs filopodia from approaching capillaries interconnect forming complex conglomerates that precede the anastomotic event. The contacting filopodia form narrow spaces between them filled with proteinaceous basal lamina material. The original narrow spaces coalesce into larger ones leading to the formation of a single one that will interconnect (anastomose) the two approaching capillaries. The four leading ECs (two for each approaching capillary) become the wall of the newly formed post-anastomotic CNS capillaries. These new CNS capillaries are very small with narrow and irregular lumina that might permit the passage of fluid but not yet of blood cells. Eventually, their lumen enlarges and permits the passage of blood cells.

## Introduction

Anastomoses of blood capillaries occur during the development of any organ or tissue, playing significant roles in the healing of traumatic and surgical wounds and in bone fractures. Despite their clinical and biological importance and widespread occurrence, some aspects in the anastomosis of blood capillaries remain inadequately studied. An important aspect in the development of blood capillaries is the role of the filopodia decorating the leading endothelial cells (ECs) of the growing capillaries. The latter explore the surroundings, determine directional growth, search for filopodia of other growing capillaries and establish complex (conglomerate) interactions leading to their anastomosis. Searching filopodia are a universal property of moving cells (Marín-Padilla, 2012).

Recently, one of us (MM-P) has explored several neuronal and microvascular aspects of the developing human cerebral cortex, using the classic Golgi method and the electron microscope. These studies include: (a) equidistant perforations (of ca. 500 μm) of the cortical glial external limiting membrane by meningeal (pial) capillaries, delineating independent vascular functional territories (Marín-Padilla, 2011a); (b) the establishment of a Virchow-Robin compartment around each perforating vessel that functions as the sole drainage system of the cortex (Marín-Padilla and Knopman, 2011); (c) the structure and development of pyramidal neurons (Marín-Padilla, 2014); and (d) the establishment of two separated blood circulations: a rich one for the gray matter, where neuron reside, and a poorer one for white matter, essentially deprived of neurons (Marín-Padilla, 2015).

In this article, we explore the anastomosis of capillaries in the central nervous system (CNS) during development and the role of the ECs filopodia in the process, using the Golgi method and the electron microscope. The cortical gray matter was selected for its rich capillarogenesis that accompanies functional maturation of its neurons (Marín-Padilla, 2012).

## Overview of the Development of CNS Capillary Anastomoses During Development

Various mechanisms have been proposed for the anastomosis of blood capillaries including: the presence of additional ECs with formation of multicellular tubes and extracellular spaces (Blum et al., 2008); the rearrangement of ECs with formation of multicellular tubes (Herwig et al., 2011); blebbing of the membranes of ECs (Gebala et al., 2016); and, the presence of additional ECs at the head of the growing capillaries, reorganization of ECs and the confluence of small fissures into larger ones (Ortuño Pacheco, 1971), Although these processes do occur in the anastomotic process, how they contribute to it remains unclear. Recent color videos in living animals have shown extraordinary images of blood vessels approaching, fusing and establishing a common (shared) lumen for the passage of blood (Lenard et al., 2015). Despite the beauty, clarity and accuracy of the videos, it was not possible to visualize vessels at the photographic magnification used. On the other hand, the possible role of the filopodia in the anastomotic process has been questioned, though admitting it might apply in some tissues (Phng et al., 2013). In our experience, as outlined below, the best method for visualizing growing capillaries and their filopodial is the Golgi method.

**Figure 1:**
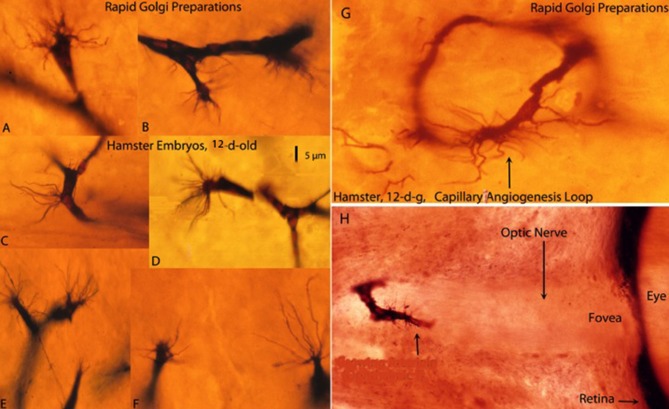
Composite figure of rapid Golgi preparations showing growing capillaries in the cerebral cortex of 12-day-old hamster embryos. Growing capillaries have a polypoid head with numerous radiating filopodia **(A,B,D–F)**. **(C)** Detail of a new capillary growing directly from the vessel wall. **(G)** Detail of a newly established capillary loop with some peripheral filopodia that have not participated in the anastomotic process. **(H)** Detail of a growing capillary (arrow) with leading filopodia entering the optic nerve of a hamster embryo’ eye. The retina and the fovea are also shown. The 5 μm scale **(D)** is roughly applicable to all capillaries of the figure excepting the eye’ one **(H)**, from Marín-Padilla (2012).

The anastomosis of CNS capillaries is a complex process involving several sequential events that include: (a) capillary approach and recognition; (b) complex filopodia interconnections (conglomerates) of the approaching capillaries with creation of narrow extracellular spaces (fissures) between them, filled with secreted basal lamina material; and (c) progressive confluence of fissures into larger ones leading to the establishment of a single cavity that will eventually become luminal and thus interconnect (anastomose) the capillaries; and, the new post-anastomotic CNS capillaries. These events are accompanied by ECs mitoses and displacements. Four extra ECs, two from each approaching capillary, move towards the capillary head and will be major contributors in the anastomotic process, being the only ones with searching filopodia (Figure 1). The meningeal capillaries that perforate the cortex external glial limiting membrane to enter into the nervous tissue also have perforating filopodia (Marín-Padilla, 2012).

## Golgi Studies of CNS Capillary Formation

It must be understood that the Golgi method is not a method in the strictest sense of the word, but a reaction. Golgi (1873) named his technique: “reazione nera” (black reaction). This silver reaction is unreliable, capricious and quite a frustrating one to use. The results depend on many factors including: kind of specimen and its age, osmic acid fixation time, silver impregnation time, specimen thickness, post-mortem interval, and others less prominent factors. It should be accepted that often it does not work. Undoubtedly, these technical difficulties explain why it has been so seldom used in research. Camilo Golgi told us that the secret of his reaction relies on: “Provando e Reprovando” (trying and trying again). As director of the Pediatric Autopsy Service (1962–1990) of the Medical School Hospital and following Golgi’s suggestion, I prepared as many Golgi preparations of developing brains as I could, hoping that in some of them the desired objective would be attained (Marín-Padilla, 2012). I made (over many years) more than 4,500 Golgi preparations of the developing mammalian brain, including man (Marín-Padilla, 2011b). This collection has been used in the present study of the anastomoses of CNS blood capillaries.

The Golgi reaction uses thick tissue preparations (150–170 μm) permitting three-dimensional visualization of neurons, neuroglia, axon terminals and growing capillaries as well as their morphological interrelationships. Despite technical difficulties, the Golgi reaction still is undoubtedly the best procedure to study the structure of the CNS, abundantly demonstrated by Cajal’ extensive work (see Ramón y Cajal, 1909, 1911, 1921). In Golgi preparations, CNS capillaries are stained black against a transparent yellowish background and measure 6–7 μm in diameter (Figures 1A–F). The advancing head of growing CNS capillaries is characterized by a polypoid bulge with numerous radiating filopodia (Figures 1A–G). The polypoid head with radiating filopodia of the growing capillaries was originally described by Klosovskii (1963), a Russian investigator, using a silver stain. They were more recently described again by Puelles et al. (1976) and, in greater detail, in the Golgi studies of Marín-Padilla (2011a) and Marín-Padilla (2012). Apparently, Ramón y Cajal (1909, 1911), drew the filopodia of growing CNS capillaries (Puelles, 2009), but interpreted them as glial processes. In a later book, “Manual de Histología Normal” (1921), Cajal described an endothelial cell filopodium as a single punctiform structure emanating from the capillary wall. We have seen similar structures, in our Golgi preparations, which we have interpreted as a reabsorbing capillary rather than a growing one.

The presence of acting filaments in the filopodia could contribute to their searching movement and advancement toward other filopodias.

The cortical gray matter displays complex short-linked anastomotic plexuses of intrinsic capillaries, between adjacent perforators, where neurons, neuroglial cells, axon terminals and capillaries reside and interact. Blood circulation through the capillary plexus between perforator vessels responds to the functional needs of its neurons mediated by the local glia (Marín-Padilla, 2012). The cortical gray was selected for the present overview because of its rich capillary plexus, where the possibility of observing capillary anastomoses is larger.

New capillaries emerge and recede from any place along the wall of existing capillaries (Figure 1C), establishing loops between different tissue levels (Figure 1G) and interconnecting them with each other forming short-linked anastomotic plexuses (Marín-Padilla, 2012). A growing capillary with filopodia entering the optic nerve of a hamster embryo’ eye was found in a previous Golgi study (Figure 1H).

## Electron Microscopic Studies of CNS Capillaries and Filopodia

The anastomoses of gray matter capillaries also have been explored using the transmission electron microscope (TEM). For the TEM studies, one of us (LH), made new recut from old tissue blocks used in a previous study of the mouse olfactory nerve (Marin-Padilla and Amieva, 1989). She also made 70 new electron microscopy (EM) thin sections (70 nm) from the developing cortex of 11.5-day-old mouse embryos, for joint examination.

In the TEM preparations, three fundamental aspects of growing capillaries in the mouse developing cortex were explored: (a) composition of the polypoid head of growing CNS capillaries nature and; (b) nature and composition of filopodial interactions (conglomerates) between approaching capillaries; and (c) the structure of new post-anastomotic. These vascular features had not yet been investigated appropriately.

**Figure 2:**
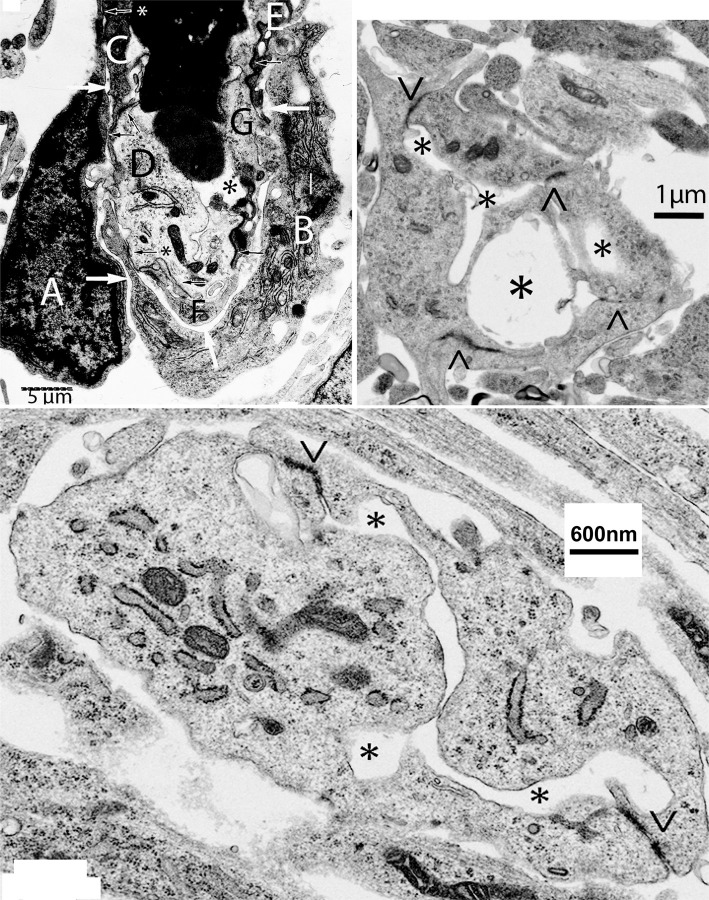
Composite figure of electron microscopy micrographs showing growing capillaries in the mouse cerebral cortex. *Top-left*, electron microscopic view of the bulging head of a perforating meningeal capillary with nucleated red cells in its lumen (asterisk), showing its endothelial cell (EC) composition and organization. Some of the ECs (C–G) are components of the capillary wall and share tight junctions (black arrows). The two additional ECs (A,B) are separated by a thin intercellular space from those of the capillary wall by a long space (white arrows) and share small tight junctions with those of the capillary wall (arrows with asterisks). The presence of two additional ECs (A,B) explains the bulging of the polypoid head of growing capillaries (see Figure 1). The additional ECs (A,B) are the only ones with searching filopodia. *Top-right*, electron microscopic view of a new post-anastomotic central nervous system (CNS) capillary composed of three ECs separated by tight junction (<, >) and with three irregular lumina (asterisks), reflecting a multiple luminal origin. *Bottom*, view of recently formed post-anastomotic CNS capillary composed of two ECs separated by tight junctions (<, >) with a small narrow and convoluted single lumen (asterisks). The post-anastomotic new CNS capillaries will permit the passage of fluid but not yet of blood cells. Eventually, their lumen will enlarge and permit the passage of blood cells.

The bulging (polypoid) head of growing CNS capillaries is composed of two types of ECs. Some ECs are standard components of the capillary wall (Figure 2, top-left, C–G) while others are extra divided ones (Figure 2, top-left A,B). The two additional ECs are separated from the capillary wall by a long narrow space (Figure 2, top-left, white arrows) but share small tight junctions with those of the wall (Figure 2, top-left, black arrows). The additional presumably ECs move toward the capillary head, contributing to its polypoid bulge and produce the filopodia (Figures 1, 2, top-left). The four newly added ECs (two from each approaching capillary) are the only ones with searching filopodia (1).

The leading filopodia of approaching capillaries establish complex interactions (conglomerates) among themselves. It is difficult to recognize these conglomerates as vascular structures. The presence of tight junctions (recognized only in high power views) among the contacting filopodia identifies them as vascular structures. Also, the presence of tight junctions among the filopodia suggests a different ECs origin, as will be those from the two approaching capillaries. Some of the filopodia conglomerates can even enclose the nucleus of one of the participant ECs. Probably, the progressive accumulation of basal lamina among the filopodia might contribute to the enlargement of the spaces between them and eventually their transformation into luminal spaces. The conglomerates have some additional peripheral filopodia that do not participate in the process.

The new post-anastomotic CNS capillaries are very small, ranging from 2.5 to 3.5 μm in diameter. Some may still have more than one lumen reflecting their multiple origins (Figure 2, top-right, asterisks). Very small ones have a narrow and irregular lumen (Figure 2, bottom). At this developmental stage, the new post-anastomotic CNS capillaries might permit the passage of fluid but not yet of blood cells. Eventually, they will enlarge and permit the passage of blood cells.

## Conclusion

In summary, filopodia of ECs seem to play a major role in the formation of CNS capillaries during development. These structures can be visualized in 3D with the Golgi method and at higher resolution with the electron microscope. Future studies with *in vivo* techniques will be needed to extend the findings summarized in this article.

## Ethics Statement

The animal studies were reviewed and approved by the Institution Ethics Committee when the original hamsters and mouse studies were done.

## Author Contributions

MM-P processed the tissue for the Golgi method, analyzed the tissue, and wrote the article. LH processed the EM preparations and sections, and took the transmission electron microscopic microphotographs.

## Conflict of Interest Statement

The authors declare that the research was conducted in the absence of any commercial or financial relationships that could be construed as a potential conflict of interest.
